# Changes in quality of life following initial treatment of oesophageal carcinoma: a cohort study from Sri Lanka

**DOI:** 10.1186/s12885-018-5106-y

**Published:** 2018-11-29

**Authors:** Ishanka Ayeshwari Talagala, Carukshi Arambepola

**Affiliations:** 1 National Programme for Prevention and Control of Non-Communicable Diseases, Ministry of Health Nutrition and Indigenous Medicine, Colombo, Sri Lanka; 2 Department of Community Medicine, Faculty of Medicine, University of Colombo, Colombo, Sri Lanka

**Keywords:** Quality of life, Oesophageal carcinoma, Tertiary prevention, Sri Lanka

## Abstract

**Background:**

Oesophageal carcinoma is one of the leading cancers in Sri Lanka. Recent advances in treatment modalities have drastically improved the survival of these patients. However, the quality of life (QoL) among the survivors needs to be reviewed in order to recognise the need for advocating more focussed rehabilitation for oesophageal carcinoma in Sri Lanka.

**Methods:**

A prospective cohort study was conducted among 51 incident cases of oesophageal carcinoma recruited consecutively from the National Cancer Institute, Maharagama. Data were collected on their QoL using EORTC QLQ-C30 and EORTC-OES18 questionnaires validated for Sri Lankan oesophageal carcinoma patients, before and one month after the completion of initial treatment. Scoring was based on the EORTC manual. Comparison of baseline and follow-up scores was done using paired t test at significance level of 0.05.

**Results:**

Response rate was 80%. The majority consisted of squamous cell carcinoma of stage IV. On a scale of 0–100, the overall QoL (mean score = 49.8; SD = 22); and role (42.2; SD = 34), physical (53.1; SD = 29), emotional (53.4; SD = 26) and social (57.2; SD = 23) functioning were relatively low at diagnosis. The scores of functioning scales further deteriorated (difference > 5 points) following the initial treatment (*p* < 0.05). Dysphagia (mean = 54; SD = 27) was the main symptom at diagnosis, which improved significantly (*p* < 0.05) in contrast to dry mouth (mean = 39.2; SD = 34) that worsened (*p* < 0.05) following initial treatment. Family support and financial difficulties were adversely affected (p < 0.05) during the initial treatment.

**Conclusions:**

The deterioration of several dimensions of QoL of oesophageal carcinoma patients following the initial treatment highlights the need for more targeted tertiary preventive strategies that address the issues identified.

## Background

Oesophageal carcinoma plays a leading role in the global cancer burden [1, 2]. It is identified as a virulent tumour particularly in the developing countries [3], thus imposing prompt action against it in these regions. Sri Lanka, a developing country in South Asia continuously reports oesophageal carcinoma as one of its five leading cancers [4]. According to the latest statistics available for Sri Lanka, 6.3% of all reported incident cancer cases were due to oesophageal carcinoma and it continues to be the third commonest cancer among males while being the sixth commonest among females [4].

Oesophageal carcinoma is known to be associated with poor prognosis, with five-year survival rates of 5–10% [5]. However, the recent advancements in treatment modalities of oesophageal carcinoma have drastically improved the survival of these patients within the last few decades around the world [6]. The situation is similar in Sri Lanka owing to the well-established free curative healthcare services in the country. Nevertheless, how improvement in survival has affected the quality of life (QoL) of the patients still remains inconclusive [7, 8].

Various treatment options available for oesophageal carcinoma may improve the survival, and thereby improve the QoL of the patients following the initial treatment. However, most patients (even with resectable oesophageal carcinoma) may have serious co-morbid conditions, thus the surgery would be poorly tolerated [9]; patients may also be affected by adverse effects following chemotherapy and radiotherapy [10]; troublesome symptoms such as dysphagia may recur [11], which all may affect the QoL of the patients with oesophageal carcinoma as a whole. Also, for some advanced stages of the disease, supportive care by means of palliation to improve their QoL, is essential [12].

The World Health Organization (WHO) defines QoL as “individuals’ perception of their position in life in the context of the culture and value systems in which they live and in relation to their goals, expectations, standards and concerns” [13]. It is a broad concept that is affected in a complex manner by a person’s physical health, psychological state, level of independence, social relationships, personal beliefs and their relationship to salient features of their environment [13]. In addition, medical parameters such as the mode of treatment, type of tumour and stage of disease [14], demographic variables [15] and the support extended by family members [16] have also shown to be important independent variables that affect the QoL of cancer patients.

Therefore, beyond survival from the disease, the important management end points would be the preservation, enhancement of patients’ independence in activities of daily living and their satisfaction. Improving QoL in cancer patients has thus become an important therapeutic goal, and is also considered as an important outcome parameter. Currently, most treatment decisions are heavily influenced by their effect on the patients’ QoL [17]. Therefore, a need arises to enhance the over-all QoL of oesophageal cancer survivors through rehabilitation initiated at the time of diagnosis and continued until the terminal stage [18].

As in many other countries, the national policy for cancer prevention and control in Sri Lanka aims at a comprehensive programme [19], which includes the provision of palliative care services through universal health coverage. Even though many initiatives have been undertaken in this regard, further improvement of these services has been hampered due to the lack of evidence on the status (either improvement or deterioration) of the QoL of patients following the initial treatment. Bridging this knowledge gap on QoL aspects in the existing cancer management would enable targeted palliative care services as a tertiary preventive strategy in the country. Therefore, this study was aimed at assessing the change of QoL of the patients with oesophageal carcinoma following the initial treatment, irrespective of the treatment modality. Findings of this study would be helpful as evidence to improve cancer management programs of similar low resource settings as well.

## Methods

A prospective cohort study was conducted during 2015–2016 period to compare the QoL of patients with oesophageal carcinoma, before and one month after the completion of initial treatment at the National Cancer Institute, Maharagama (NCIM). The NCIM is the only tertiary referral hospital situated in the Western province of Sri Lanka for cancer care. This state hospital is dedicated for free of charge in-ward and out-patient cancer care services including chemotherapy, radiotherapy and follow-up of cancer patients referred from both state and private sector hospitals. It caters mainly for the residents of Western province, which is the most populated province in Sri Lanka. Study participants were residents in the Western province, who were newly diagnosed with oesophageal carcinoma based on the histological confirmation following upper gastrointestinal endoscopy (UGIE) examination done within the last three months, and in whom the initial treatment (surgery, chemotherapy and/or radiotherapy) has not commenced by the date of recruitment into the study. Critically ill patients, patients with secondary carcinoma (e.g. metastasis) including oesophageal carcinoma, patients diagnosed with any other cancer (confirmed with documental evidence) and patients with relapses of any cancer including oesophageal carcinoma were excluded from the study.

Based on the available data for cancer incidence in Sri Lanka, Western province continuously reports very high incidence of Oesophageal carcinoma [20, 21]. Nevertheless, Western province being the most populated province of Sri Lanka, consists of the highest socio-economic diversity and include people from urban, rural and estate sectors, belonging to all social classes [22]. Therefore, findings of the quality of life of patients with oesophageal carcinoma is assumed not to differ much from the rest of the country. Thus, the current study was conducted in the Western province of Sri Lanka.

The sample size of the study was calculated with the aim of detecting clinically significant changes (either increase or decrease) in the QOL of patients with oesophageal carcinoma after the initial treatment. Thus, detecting at least a change between 5 and 10% in the QOL core questionnaire [23] was aimed at. This change is known to be noticed by the patients as a ‘little’ better or worse change and is regarded as the smallest clinically detectable significant change in QOL assessment [24].

Therefore, the calculated sample size of 51 was based on 5% significance level, beta error of 0.2 and 25% non-response rate, to detect at least a ‘little’ change in the QoL (mean difference = 3.2; standard deviation = 7.7) between pre-treatment and post-treatment scores [24] following the initial treatment among patients with oesophageal carcinoma.

Following informed written consent, patients were consecutively recruited from the oncology and surgical wards and clinics, and were administered an interviewer-administered questionnaire to obtain data on socio-demographic characteristics. The QoL of participants was assessed using three questionnaires: EORTC QLQ-C30 core questionnaire and EORTC QLQ-OES18 module developed by the European Organization for Research and Treatment of Cancer (EORTC) – quality of life group [23], and another questionnaire on family support scale. Since the QLQ-C30 has been designed to assess only the generic aspects of QoL of patients with cancer, the QLQ-OES18 which is on the disease and treatment specific QoL of patients with oesophageal carcinoma was adopted to supplement the QLQ-C30 [25]. The family support scale was a locally developed questionnaire. All these questionnaires had been previously validated by Jayasekara (2006) for Sri Lankan adult patients with oesophageal carcinoma [8].

The EORTC QLQ-C30 (version 3.0) questionnaire incorporates nine multi-item scales: five functional scales (physical; cognitive; emotional; and social functioning); three symptom scales (fatigue; pain; and nausea and vomiting); and one scale on global health status/quality of life [26]. The EORTC QLQ-OES18 comprises four scale items (dysphagia; problems with eating; reflux; and pain) and six single items (trouble swallowing saliva; choked when swallowing; dry mouth; trouble with taste; trouble with coughing and trouble talking) [27]. The family support scale includes three items (whether the patient felt lonely; unhappy with the attention paid by the family; unhappy with the support extended by the family) [8].

The QoL of each patient in the cohort was assessed at two time points; initially at the time of diagnosis (base-line data) prior to their initial treatment, irrespective of the treatment plan. After obtaining their basic characteristics, the QLQ-C30 core questionnaire, family support scale and the QLQ-OES 18 were administered in sequence. The same cohort was then followed up to re-assess their QoL one month after completion of their initial treatment (follow-up data). Since the QoL of patients is well known to deteriorate during the acute phase of treatment due to its side effects [28, 29] and also soon after the initial treatment [8], it was decided to give a one month of treatment free period following the initial treatment, with the assumption that the side effects would wear off by then.

Data were analysed using the statistical package for the social sciences (SPSS) version 20. The scoring for the questionnaires were given based on the EORTC scoring manual, and all scale and single item scores were linearly transformed to a 0–100 scale [30]. Standardized skewness was calculated (to check whether it falls within − 3.0 to 3.0) wherever necessary prior to the application of parametric analytical tests [31] . Scores of the follow-up study were compared with the baseline scores of QoL using paired t test at a significance level of 0.05.

## Results

The sample consisted of 51 newly diagnosed patients with oesophageal carcinoma, with ages ranging from 37 to 79 years. The mean age of the study participants was 60.2 (SD = 11.2) years with the median being 60.0 (IQR = 53.0–69.0) years. Majority of them were males (70.6%). The basic characteristics of the patients are given in Table 1.

**Table 1:** Basic characteristics of the patients with oesophageal carcinoma

Characteristic	No. (*N* = 51)	%
Sex		
Male	36	70.6
Female	15	29.4
Age		
30–39	2	3.9
40–49	7	13.7
50–59	15	29.4
60–69	16	31.4
70–79	11	21.6
Current civil status		
Married	43	84.3
Unmarried/widowed	8	15.7
Highest educational qualifications		
Primary and lower	16	31.4
Secondary education	34	66.6
Tertiary education	1	2.0
Employment status		
Currently employed	31	60.8
Previously employed	13	25.5
Never employed	7	13.7
Social status^a^		
Social class I – Leading professions	1	2.0
Social class II – Lesser professions	2	3.9
Social class III- Skilled workers and non-manual workers	15	29.4
Social class IV – Partly skilled workers	15	29.4
Social class V- Unskilled workers	18	35.3
Monthly family income (USD)^b^		
< 126	25	49.0
127–315	21	41.2
316–630	5	9.8

^a^Classification based on the occupation

^b^1 USD = LKR 158.73

Majority of the patients were diagnosed with squamous cell carcinoma (63%) and in advanced stage (56.2%). Among the 14 patients who underwent surgery as their initial treatment modality for oesophageal carcinoma, 10 (71.4%) underwent oesophagectomy while the rest (28.6%) underwent stenting. Their clinical characteristics are compared with adenocarcinoma at the time of diagnosis (Table 2).

**Table 2:** Clinical characteristics of the patients with oesophageal carcinoma at the time of diagnosis

Characteristic	Squamous cell carcinoma (*n* = 32)		Adenocarcinoma (*n* = 19)	
	No.	%	No.	%
Grade of the tumour				
Well differentiated/G1	5	15.6	1	5.3
Moderately differentiated/ G2	21	65.6	12	63.2
Poorly differentiated/ G3	6	18.8	6	31.6
Stage of the tumour				
Stage I	0	0.0	1	5.3
Stage II	2	6.2	7	36.8
Stage III	12	37.6	7	36.8
Stage IV	18	56.2	4	21.1
Initial treatment undergone				
Surgery	6	18.8	8	42.1
Radiotherapy	10	31.2	2	10.5
Chemotherapy	4	12.5	5	26.3
Neo-adjuvant chemo-radiotherapy	11	34.4	2	10.5
No treatment	1	3.1	2	10.5
Time since first symptom to diagnosis				
< 3 months	20	62.5	13	68.5
> 3 months	12	37.5	6	31.5
Long standing co-morbid conditions				
Yes	2	6.3	3	15.8
No	30	93.7	16	84.2

Of the 51 patients enrolled for follow up (*n* = 51), 10 (19.6%) died subsequently during the follow-up and therefore, the comparison of QoL scores between the baseline and follow-up was done among 41 patients. Among those who died, 50% were of more than 60 years in age; 90% were of low educational level (up to secondary level); 80% belonged to social class III, IV and V; and 80% had metastasized disease. The average duration between baseline assessment of the QoL and the follow up assessment for the patients who underwent surgical treatment, radiotherapy, chemotherapy and neoadjuvant chemo-radiotherapy was 2.3 months (SD = 1.2); 3.8 months (SD = 2.3); 5.2 months (SD = 2.8) and 4.2 months (SD = 1.8) respectively. It should also be noted that the follow up assessment was done following a one month of treatment free period, after completing the initial treatment.

Table 3 shows the changes in the generic dimensions of QoL of patients before and after the initial treatment. The global health status/QoL of the patients has deteriorated between the baseline and follow-up following the initial treatment by 8.33 (SD = 30; 95% CI: -18.0, 16.7). However, this difference was not significant (paired *t* = 1.8; df = 40; *p* = 0.08). All functioning dimensions (*p* < 0.05) except cognitive functioning; and family support (*p* = 0.002) significantly deteriorated during the follow-up period. Among the general symptoms, fatigue; nausea and vomiting; pain; insomnia; loss of appetite; and constipation significantly worsened following the completion of initial treatment (p < 0.05). In addition, financial difficulties significantly worsened during the follow-up (*p* < 0.001).

**Table 3:** QOL scores for functioning and general symptoms dimensions of oesophageal carcinoma, before and after the initial treatment

Scale	Baseline score		Follow up score		Mean difference^d^ (*N* = 41)	SD	95% CI	Significance^e^
	Mean (*N* = 51)	SD	Mean (*N* = 41)^c^	SD				
Global health status/QOL	49.8	22.0	43.9	30.4	−8.33	30.0	−18.0, 1.1	*t* = 1.8; df = 40 *p* = 0.08
Physical functioning	53.07	28.8	34.31	24.5	−20.81	29.2	−30.03, −11.59	*t* = 4.56; df = 40 *p* < 0.001
Role functioning	42.16	34.5	21.54	26.2	−22.76	37.8	−34.69, −10.84	*t* = 3.86; df = 40; *p* < 0.001
Emotional functioning	53.43	26.4	42.07	31.2	−14.03	32.8	−24.38, −3.69	*t* = − 2.74; df = 40 *p* = 0.009
Cognitive functioning	75.49	23.4	71.54	26.9	−6.1	26.0	−14.31, + 2.12	*t* = 1.5; df = 40 *p* = 0.141
Social functioning	57.19	23.2	37.4	26.8	−23.17	27.9	−31.96, −14.38	*t* = 5.33; df = 40 *p* < 0.001
Family support^a^	82.57	20.4	80.49	14.4	−6.5	12.9	− 10.58, − 2.43	*t* = 3.23; df = 40 *p* = 0.002
Fatigue	52.07	28.9	71.27	28.8	20.87	35.2	9.78, 31.96	*t* = 3.8; df = 40 *p* < 0.001
Nausea and vomiting	40.2	34.4	54.07	37.6	13.0	39.0	0.7, 25.31	*t* = 2.14; df = 40 *p* = 0.039
Pain	45.42	32.7	71.54	28.7	27.64	37.6	15.78, 39.5	*t* = 4.71; df = 40 *p* < 0.001
Dyspnoea	24.18	26.7	27.64	29.7	5.69	30.6	−4.0, 15.36	*t* = 1.19; df = 40; *p* = 0.241
Insomnia	33.33	32.0	43.9	35.3	13.82	36.5	2.3, 25.34	*t* = 2.43; df = 40 *p* = 0.02
Loss of appetite	42.48	34.0	54.47	37.8	13.0	40.1	0.37, 25.65	*t* = 2.08; df = 40 *p* = 0.044
Constipation	30.06	35.4	47.97	41.5	16.26	34.3	5.45, 27.07	*t* = 3.04; df = 40 *p* = 0.004
Diarrhoea	10.46	18.2	4.88	14.1	−5.69	21.0	−12.3, 0.92	*t* = 1.74; df = 40 *p* = 0.09
Financial difficulties^b^	50.98	31.5	77.24	21.6	30.89	32.8	20.53, 41.25	*t* = 6.03; df = 40 *p* < 0.001

^a^Scores range from 0 to 100, with higher scores indicating a higher level of functioning/family support

^b^Scores range from 0 to 100, with higher scores indicating a higher degree of financial difficulties

^c^Ten patients died during the follow up period

^d^A negative mean difference indicates deterioration of functioning, family support and improvement of symptoms and a positive mean difference indicates worsening of symptoms and financial difficulties

^e^paired t test

Changes in the disease-specific dimensions of QoL before and after the initial treatment are given in Table 4. Among the disease-specific symptoms, dysphagia significantly improved following the initial treatment of oesophageal carcinoma (mean difference = − 13.6; SD = 29.7; *p* = 0.006); while dry mouth significantly worsened (mean difference = 20.3; SD = 39.4; *p* = 0.002). Improvement of trouble swallowing saliva and choking when swallowing, and the worsening of other symptoms following the initial treatment however were not statistically significant (*p* > 0.05).

**Table 4:** QOL scores for disease specific dimensions of oesophageal carcinoma, before and after the initial treatment

Disease-specific dimension^a^	Baseline score		Follow up score		Mean difference^c^ (*N* = 41)	SD	95% CI	Significance^d^
	Mean (*N* = 51)	SD	Mean (*N* = 41)^b^	SD				
Dysphagia	54.03	27.0	37.13	29.0	−13.55	29.7	−22.91, −4.19	*t* = 2.93; df = 40 *p* = 0.006
Problems with eating	31.21	22.0	30.28	23.1	1.63	27.3	−7.0, 10.25	*t* = 0.38; df = 40 *p* = 0.705
Reflux	42.48	29.5	44.97	36.4	7.32	36.9	−4.33, 18.97	*t* = 1.27; df = 40 *p* = 0.212
Pain	29.63	25.7	30.08	32.3	3.25	32.8	−7.1, 13.6	*t* = 0.64; df = 40 *p* = 0.529
Trouble swallowing saliva	24.84	35.2	22.76	32.9	−2.44	34.5	−13.32, 8.44	*t* = 0.45; df = 40 *p* = 0.653
Choked when swallowing	22.88	30.2	18.7	26.9	−1.63	30.7	−11.31, 8.06	*t* = 0.34; df = 40 *p* = 0.736
Dry mouth	39.22	34.4	58.54	37.1	20.33	39.4	7.9, 32.74	*t* = 3.31; df = 40 *p* = 0.002
Trouble with taste	25.49	32.4	37.4	35.1	13.0	46.5	−1.66, 27.68	*t* = 1.79; df-40 *p* = 0.081
Trouble with coughing	37.25	32.4	42.28	39.5	7.32	36.9	−4.33, 18.96	*t* = 1.27; df = 40 *p* = 0.212
Trouble talking	32.68	31.6	28.46	33.8	0.0	41.5	−13.1, 13.1	*t* = 0.0; df = 40 *p* = 1.00

^a^Scores range from 0 to 100, with higher scores indicating a higher degree of symptoms

^b^Ten patients died during the follow up period

^c^A positive mean difference indicates worsening of symptoms and negative mean difference indicates improvement of symptoms

^d^paired t test

## Discussion

Our study, which aimed at assessing the changes in QoL of the patients with oesophageal carcinoma following the initial treatment, highlights that the generic as well as disease-specific dimensions of the QoL changes with time. In particular, the physical, role, emotional and social functioning, and general symptoms such as fatigue, nausea and vomiting, pain, insomnia, loss of appetite and constipation are significantly worsened during the follow-up. Among the disease-specific dimensions, dysphagia showed a significant improvement following the initial treatment in contrast to dry mouth. However, the deterioration of the overall global health status/QoL of the patients was found to be non-significant.

Quality of life is already considered an important indicator of the quality of cancer care, especially in developed countries, where most cancer treatment decisions are based not only on their effects on patient survival but also on the QoL [17]. Such decision making is not yet an integral component in the cancer management protocols in many developing countries, including Sri Lanka, mainly due to the lack of evidence on the QoL of patients with oesophageal carcinoma following treatment. Moreover, such evidence from developed countries may not be well applicable in the developing countries, given the low resource settings and socio-cultural differentials in these countries.

An important finding of the current study is that the patients with oesophageal carcinoma have a low mean score for global health status/QoL (mean score = 49.8; SD =21.9) at the time of diagnosis. This finding is compatible with another study conducted in Sri Lanka (mean score = 47.9; SD = 22.8) [8] and with the reference value given for baseline in the EORTC (mean score = 55.6; SD = 24.1) [32]. Mental and physical burden of the patients following the disease process, diagnosis and social stigma associated with more culture-specific factors, could be the reasons following this low overall QoL that persists from the time of diagnosis among the patients, irrespective of how advance the disease is. This situation is likely to have been worsened by the majority of patients being 60 years of age and above (53%); of low educational level (67% educated up to secondary level) and low social status (social class IV and V); with suffering from the symptoms of the disease for more than 3 months (69%) and with metastatic disease (43%).

The unpleasant acute adverse effects of cancer treatment, such as lethargy, alopecia, nausea and vomiting, mucositis and diarrhoea are known to be short-lived, which usually wear off by two-three weeks following therapy [33]. This justifies the assessment of QoL after one month treatment free period in the current study, so that such acute effects were minimal on their QoL [33]. Nevertheless, the present study shows that the global health status, the functioning dimensions of the QoL and the general symptoms of cancer deteriorate from the baseline, indicating that there are many factors other than the stage of the disease [12] and the treatment modality, that affect the QoL of patients with oesophageal carcinoma. Therefore, findings seen on the change in QoL following the initial treatment may not truly reflect the sole effect of the treatment modality on the QoL of the patients in Sri Lanka. Thus, other potential influencing factors need to be further explored preferably using qualitative research methods.

The current study importantly points out a significant improvement in dysphagia, the major symptom related to oesophageal carcinoma, following the initial treatment. However, dry mouth, loss of appetite and nausea and vomiting were significantly worsened during the follow up. Though not statistically significant, trouble with taste also was worsened during the follow up. All these factors may have collectively resulted in the worsening of problems with eating among the patients with oesophageal carcinoma, although dysphagia was significantly improved following the initial treatment. Oesophageal carcinoma is a common disease to lose weight due to its disease process. The anthropometry measurements in the current study were taken at the time of recruitment of the patients to the study, after the onset of the disease, by which time a higher proportion of patients had lost their weight. Therefore, interpreting the findings of the study utilizing the current weight of the pateints will not portray the correct improvement or deterioration of body weight following the increased or decreased ability to consume meals by the patients after the initial treatment. Thus, the authors did not include the body weight in the manuscript.

Pain, fatigue and insomnia are also not alleviated by the initial treatment modalities. Though not statistically significant, many other disease specific symptoms were worsened during the follow-up as well. This suggests the need of incorporating therapeutic interventions into the rehabilitation program of the country to counter act these. However, Jayasekara (2006) cites that the interpretation of the results on reflux has to be done cautiously as there are existing doubts regarding the ability of the scale to measure this aspect [8]. Also, there are doubts whether reflux is a co-morbidity or a consequence of the oesophageal carcinoma and its treatments [8].

In the present study, significant deterioration of the physical, role, emotional and social functioning was seen after completion of the initial treatment, which was comparable with another study conducted in Sri Lanka [8]. However, the current study failed to detect a significant change in the cognitive functions following the treatment. In contrast, Jayasekara (2006) found a marginal improvement in the cognitive functioning from the baseline. He further reported that based on the validation results of QLQ-C30 to Sri Lankan oesophageal carcinoma patients; there is a further need to incorporate additional items to cover several aspects of cognitive functioning in the original questionnaire [8]. Thus, the interpretation of cognitive functioning scale scores has to be done cautiously.

Another noteworthy finding of this study is the significant worsening of financial difficulties between the baseline and the follow-up, reflecting the impact of the disease and treatment on the economical aspect of the patient. Even though higher baseline mean score was obtained for the family support reflecting the support extended by the family during the illness, this significantly deteriorated during the follow-up. Given the low health spending of 2% Gross Domestic Product in 2014 [34], cancer care in Sri Lanka is being increasingly financed by out-of-pocket expenditure [35]. This financial burden incurred on the family who are the care givers of the patients could have resulted in the deterioration of family support with time. Additional costs on adopting newer technologies in their treatment would have worsened this matter. This implicates the importance of harnessing social support and protection, and financial protection for the patients as integral parts of universal health coverage, especially in developing countries like Sri Lanka.

In summary, the significant reduction observed in the functioning of patients resulting in low level of independence; worsening of debilitating symptoms such as fatigue, pain, insomnia, loss of appetite, nausea/vomiting, dry mouth, trouble coughing, reflux and problems with eating; no improvement in trouble with talking resulting in social isolation; worsening of financial difficulties and reduction of family support, all would have a cumulative effect on the QoL of patients with oesophageal carcinoma. Therefore, all these findings should be taken into consideration when planning and implementing palliative care activities for patients with oesophageal carcinoma in Sri Lanka.

### Strengths and limitations

The current study included patients of varying educational status. Therefore, even if the questionnaires used had been validated as self-administered ones, these were administered by interviewers in the present study. This could have resulted in over or under estimation of this assessment.

The current study was conducted in the Western province of Sri Lanka. Since the NCIM is the only tertiary care referral hospital for cancer care in the Western province dedicated to provide free of charge cancer management services, oesophageal carcinoma patients diagnosed both from other state and private sector hospitals are referred to NCIM for further management. Thus, the current study sample is well representative of the oesophageal cancer patients in the Western province. However, authors are aware that one may argue with the generalizability of the current study findings to oesophageal cancer patients in Sri Lanka. Western province has continuously reported high incidence of oesophageal carcinoma. Also, it is the most populated province of Sri Lanka and consists of the highest socio-economic diversity and include people from urban, rural and estate sectors, belonging to all social classes. Therefore, findings of the quality of life of patients with oesophageal carcinoma is assumed not to differ much from the rest of the country. Nevertheless, there is a possibility that the QoL of oesophageal carcinoma patients from rural areas of Sri Lanka be worse than that is presented in the current study.

The objective of the current study was to assess the QoL of patients with oesophageal carcinoma following the initial treatment, irrespective of the treatment modality. Therefore, a detailed analysis of how much the QoL varies specific to a certain treatment modality was not assessed.

## Conclusions

The QoL of patients with oesophageal carcinoma was shown to be multi-dimensional. Further deterioration seen of physical, role, emotional and social functioning; general and disease-specific symptoms following the initial treatment emphasizes the need for incorporating treatment and management options in the rehabilitation program. Worsening financial difficulties and the decline in family support reveal the need for counselling of patients and their family members, and the importance of financial protection of patients, as an integral part of the universal coverage of services to cancer patients in Sri Lanka. These findings emphasise the importance of the implementation of community based rehabilitation programmes through palliative care approach by the state for the tertiary prevention of oesophageal carcinoma by improving the QoL of patients with oesophageal carcinoma.

## Abbreviations

DALY: Disability Adjusted Life Years
EORTC: European Organization for Research and Treatment of Cancer
NCIM: National Cancer Institute Maharagama
QoL: Quality of life
SPSS: Statistical Package for the Social Sciences
UGIE: Upper gastrointestinal Endoscopy
WHO: World Health Organization
